# Gut microbiota development in the growing dog: A dynamic process influenced by maternal, environmental and host factors

**DOI:** 10.3389/fvets.2022.964649

**Published:** 2022-09-02

**Authors:** Quentin Garrigues, Emmanuelle Apper, Sylvie Chastant, Hanna Mila

**Affiliations:** ^1^NeoCare, ENVT, Université de Toulouse, Toulouse, France; ^2^Lallemand Animal Nutrition, Blagnac, France

**Keywords:** microbiome, puppy, canine, bacteria, health, growth, nutrition, treatment

## Abstract

Microorganisms of the gastrointestinal tract play a crucial role in the health, metabolism and development of their host by modulating vital functions such as digestion, production of key metabolites or stimulation of the immune system. This review aims to provide an overview on the current knowledge of factors shaping the gut microbiota of young dogs. The composition of the gut microbiota is modulated by many intrinsic (i.e., age, physiology, pathology) and extrinsic factors (i.e., nutrition, environment, medication) which can cause both beneficial and harmful effects depending on the nature of the changes. The composition of the gut microbiota is quickly evolving during the early development of the dog, and some crucial bacteria, mostly anaerobic, progressively colonize the gut before the puppy reaches adulthood. Those bacterial communities are of paramount importance for the host health, with disturbance in their composition potentially leading to altered metabolic states such as acute diarrhea or inflammatory bowel disease. While many studies focused on the microbiota of young children, there is still a lack of knowledge concerning the development of gut microbiota in puppies. Understanding this early evolution is becoming a key aspect to improve dogs' short and long-term health and wellbeing.

## Introduction

Nowadays, gut microbiome is considered as the equivalent of a new organ, pivotal for the survival of the host (1). Indeed, the microbiota of the gastrointestinal tract (GIT) is a highly complex structure composed of trillions of microorganisms depending on the species. For example, there are about 10^10^ bacteria in just 1 ml of cow's rumen (2), while there are about 10^13^ microorganisms in total in the gut of omnivorous like humans and carnivorous like dogs, mainly bacteria, but also archaea, viruses, and fungi (3, 4). Those microorganisms share a deep bond with their host by offering metabolic properties that the host organism alone could not effort, such as nutrient assimilation, development of the immune system, and production of key biocompounds like vitamins contributing to the general homeostasis (5–7). Advances in DNA sequencing and biotechnology allowed to dress a precise overview of the gut microbial population and their biological activities (8). In this context, the gut microbiome quickly became a key target in research to better understand digestive health and its impact in the general health.

The GIT microbiota composition can be affected by many factors, like age, nutrition and environment (9, 10). Some of the changes induced by these factors will be followed by beneficial effects on the gut health of the host, but others can lead to shifts from a microbial equilibrium (eubiosis) to disbalance (dysbiosis), and in consequence gastrointestinal disorders (i.e., inflammatory bowel diseases) or even systemic metabolic or autoimmune diseases (10–14). The growing period, crucial in the health and development of juveniles (15), is also a critical window for microbiota colonization. During this period, the gut microbiome is even more sensitive to potential disruptors than during adulthood, and shifts in the microbiota composition occurring through this maturation period can induce health disorders later in life (16).

While the understanding of the factors involved in shaping the human gut microbiome is rapidly progressing, there is still a lack of knowledge when it comes to dogs. Despite puppies are extremely vulnerable during the first months of life, with a high pre-weaning mortality rate [about 10% of puppies born alive (17)], and a high frequency of diarrhea episodes [with about 25% of puppies affected between 5 and 14 weeks (18)], literature is actually limited when investigating the microbial communities of puppies and the potential link to digestive disorders. Due to growing evidence that intestinal microbiome plays an important role in neonates' health in different species, identification of factors influencing it, and in turn the general health in puppies, is a promising topic of research to decrease the morbidity and mortality in the canine species.

The current review aims to provide an overview of the development of the gut microbiota during the early stages of canine life, and to determine which factors play a role in the modulation of microorganisms' communities, with a potential impact on the health since birth until the adulthood. The term “puppy” used in this review refers to any dog from the age of 0 to 12 months, duration required for most puppies to reach adulthood (19, 20). The following keywords were used to establish the references's list: “puppy,” “microbiota,” “dog,” “canine,” “bacteria,” “microbiome” and “health” with the use of Google Scholar, PubMed, PMC and Web of Science search engines.

## Definition and role of microbiota

In this review, we consider the term “microbiota” as referring to the collection of microorganisms in an ecosystem (i.e., the GIT), while “microbiome” will be used when genetic elements and functions are also considered (21). The gut microbiome contributes to various metabolic functions, such as protection from pathogens, production of short-chain fatty acids (SCFAs) or education of the immune system. Those metabolic functions can have a direct impact on the dog physiology, which itself will provide proper environment for the bacteria, creating a symbiotic relationship between the gut microbiota and its host (22). Abundances of the different taxa differ along the tract of the GIT, depending on the consumption and production of metabolites (23). The GIT microbiota of dogs is commonly explored through fecal samples, which are less invasive to obtain than collecting GIT content samples, but provide less precise information of the bacterial communities such as mucosa-adherent bacteria. Analyses of the gut microbiota through intestinal segments is possible, but requires either complex chirurgical acts or euthanized animals (23–25), which in both cases, limit the panel of animals available. However, analyses of fecal samples, have allowed to detect consistent key bacterial species in healthy dogs, suggesting the existence of a core microbiota (26). Whether studying adult dogs or puppies, this core bacterial profile is composed of five main phyla: Firmicutes, Fusobacteria, Bacteroidetes Proteobacteria, and Actinobacteria (Table 1) (25, 38–42). In other mammal species such as humans, mice and pigs, Firmicutes and Bacteroidetes also represent the two most predominant known phyla (43). Dogs also share a similar relative abundance of Proteobacteria and Actinobacteria with humans and mice, but they remain the only of the three species with a high abundance of Fusobacteria. This phylum is barely present in other species, and is even associated with colorectal cancer risk in humans (44), but seems to be present in high abundance in healthy dogs, making it a specific characteristic of the dog's gut microbiota.

**Table 1:** Major functions of the five main phyla of the growing dog gut microbiota.

**Phylum**	**Major genera**	**Known functions in the gut**
Firmicutes	Clostridium, Lactobacillus, Streptococcus, Faecalibacterium, Staphylococcus, Ruminoccocus, Eubacterium	Produce various metabolites (vitamins, SCFAs, secondary bile acids) *via* carbohydrate fermentation. *Lactobacilli can ferment milk oligosaccharides and* produce acetate and lactate. Other Firmicutes (*Faecalibacterium, Eubacterium, Roseburia*) will use these metabolites to produce butyrate, serving as energy for enterocytes, providing anti-inflammatory protection (27) or limitating the colonization of pathogens, like *Salmonella* (28). *C. perfringens* and *Staphylococcus aureus* are strict pathogens.
Bacteroidetes	Bacteroides, Flavobacterium, Sphingobacterium, Prevotella	Similar activities as Firmicutes, with consumption of dietary fibers and complex polysaccharides to produce metabolites (bile acids, butyrate, vitamins, SCFAs). They also degrade glycans coming from mucin secretions, helping the host to gain energy from non usable carbohydrate sources (29). They are known to reduce intestinal oxygen level and promote the growth of strict anaerobic bacteria (30).
Proteobacteria	Escherichia, Helicobacter, Campylobacter, Proteus	Proteobacteria have many roles in protein, carbohydrate and vitamin metabolism, but, as aerobic facultative members of the GIT, their main utility appears to be the maintenance of an anaerobic environment of the gut for normal microbiome function (31). Many Proteobacteria, such as *E. coli, Salmonella, Campylobacter* and *Helicobacter* may induce dysbiosis and inflammatory disorders.
Fusobacteria	Fusobacterium, Cetobacterium, Streptobacillus	Fusobacteria have little fermentative ability. Little is known about the function of Fusobacteria in dogs. They are more abundant in obese dogs (32), but less abundant in dogs with acute diarrhea (33). Also, some Fusobacteria are able to degrade protein and amino acid and to produce SCFAs, suggesting a role in meat degradation (34).
Actinobacteria	Bifidobacterium, Corynebacterium, Collinsella	The most well-known Actinobacteria are *Bifidobacterium*, which are homo—or heterolactic fermentative, involved in the degradation of milk oligosaccharides to produce lactate and acetate (35). Higher abundance of Actinobacteria has been observed in adult obese dogs, probably due to their role in the production of energetic SCFAs (36). Higher abundance of *Collinsella* also seems to rise risks of gastric dilatation-volvulus (37).

### Firmicutes

Firmicutes is one of the top three most abundant phylum of the gut microbiota, with a high diversity of species. Among Firmicutes, Clostridia represents one of the most diverse and abundant taxa, representing 10 to 40% of the total bacteria sequenced, with a grand variety of roles (45). One of their main function is the production of butyrate in the gut. Butyrate is used as a source of energy by colonocytes that oxidize it into carbon dioxide, and render the epithelium hypoxicserving (28, 46). Some *Clostridium* species can have detrimental effects, such as *Clostridium difficile* and *Clostridium perfringens* that produce toxin plasmids and induce a lower gut microbiota diversity, facilitating the colonization of potential pathogenic bacteria (47, 48). Another important class of Firmicutes is Bacilli, mostly consisting of the genera *Lactobacillus* and *Streptococcus. Lactobacillus* produce lactate and acetate, are able to stimulate immune function and play an important role in the antigen tolerance (25).

### Bacteroidetes

The second most predominant phylum in dogs is Bacteroidetes. Most *Bacteroidaceae* are obligate anaerobes and need to wait for aerobic bacteria to consume oxygen before being able to colonize the gastrointestinal tract. In humans, this makes the appearance of Bacteroidetes to be considered as a biomarker of gut microbiota maturity (49). Bacteroidetes are able to use various types of substrates for fermentation (among which proteins and various carbohydrates, including milk oligosaccharides) and a decreased abundance of this phylum was observed in dogs with inflammatory bowel disease (50, 51). The most abundant genera of this phylum are *Bacteroides* and *Prevotella* (26). In the human gut, *Bacteroides* use glycans to interact with the gut tissue, providing protection from pathogens and supplying nutrients to the rest of the bacteria from the gut (52).

### Fusobacteria

Unlike humans, Fusobacteria is one of the three predominant phyla composing the gut microbiota in adult dogs, along with Firmicutes and Bacteroidetes, more specifically the *Fusobacterium* genus, representing around 20% of the total relative abundance (24). While it is associated with gastrointestinal disease in humans, this phylum is commonly observed in healthy dogs (53). Also, because *Fusobacterium* species were found in higher abundances in dogs and cats than in humans (54), and due to their ability to degrade proteins into amino acids and peptides (34), it is assumed that Fusobacteria are key bacteria in the gut metabolism of carnivorous animals (55).

### Proteobacteria

Despite a diverse phylum, Proteobacteria is infamously known for including opportunistic pathogens, like *Escherichia coli, Salmonella*, and *Campylobacter*, with potential impact on the health of the host. While it is true increased abundances of Proteobacteria have been associated with dysbiosis and inflammatory disorders (50, 56), those bacteria have also been shown to be present in high abundance in healthy dogs (27, 57). Proteobacteria encode a variety of functions, including protein, carbohydrate and vitamin metabolism, but, alike Bacteroidetes, their main function appears to be the ability to maintain an anaerobic environment in the gut for normal microbiome function (31).

### Actinobacteria

Actinobacteria, is the least abundant phylum in dogs, representing around 4% of the adult dog microbiota. The relative abundance of this phylum is even less abundant in puppies, with studies finding <1% of Actinobacteria in feces of puppies younger than 56 days (39). One important genus of this phylum is *Bifidobacterium*. In humans, *Bifidobacteria* are one of the first colonizers of the infant gut, playing a pivotal role in systemic and mucosal immunity of the host, as well as in milk oligosaccharides degradation (58, 59). While this family was observed in puppies from 1 to 7 weeks of age, it was not detected in any older dogs, suggesting it to be a specific bacteria of puppies' gut (40).

## Modification of the microbiota over the stages of life

Among the many factors driving the gut microbiota, age has one of the highest impact on the microbial composition (36). The development of the gut microbiome starts right at birth (if not even during fetal life) and its composition keeps evolving following the different stages of its host life. In human medicine, it was reported that most of the gut bacterial strains remain stable for decades (60). This makes the early colonization a crucial step for the newborn, as the first bacteria established will possibly shape the host gut functions for most of its life (61, 62).

### Pre-natal exposure and gut colonization at birth

Determining the exact start of the inoculation of the gut with microorganisms is still a matter of debate among researchers due to challenges in reliable sampling during gestation and low-abundance microbiota at birth. It was initially admitted that the GIT of mammals is sterile during the intra-uterine fetal life, with the inoculation of microorganisms occurring through contact with the mother's vagina, skin and ingestion of milk within the first hours following parturition (63). Known as the “sterile womb paradigm”, this assessment was recently challenged due to the emergence of molecular techniques allowing detection of bacteria in placenta, uterus or amniotic fluid in different mammals, with transmission of bacteria from the mother to the fœtus potentially *in utero* (35, 64, 65).

The possibility of intrauterine bacterial colonization of the fetus in dogs has been explored by analyzing the microbiota composition of meconium and placenta samples: bacteria were detected in 86.5% of meconium samples and 57% of placenta samples, collected immediately after birth (38). In puppies, as in humans, *Staphylococcus spp*., *Streptococcus spp*. and *Neisseria zoodegmatis*, respectively from the Firmicutes and Proteobacteria phyla, were the most common bacteria isolated from both meconium and placenta (66, 67). Interestingly, Staphylococcus appears to be one of the most common genera in the endometrium microbiota of dams, while Streptococcus is more present in their vagina, corroborating that the meconium microbiota of puppies born *via* vaginal delivery resembles partially the one of their mother's vagina and supporting a potential transplacental transfer of microorganisms (68). Indeed, a presence of microbiota, most frequently *Acinetobacter spp*., *Staphylococci* and *Bacillus spp*., in the amniotic fluid and the meconium was observed in puppies born *via* cesarean section (69).

Those results in favor of an intra uterine bacterial transfer from dam to fetus have to be interpreted with caution. Due to low concentration of bacteria, culture-based techniques can fail to identify most of the organisms and environmental contamination cannot be fully ruled out when collecting newborn samples at birth (70, 71). For those reasons, the intra-uterine bacterial transfer still remains a subject of debate.

### Colonization during the neonatal period

Following birth, the newborn gastrointestinal tract is quickly colonized by microorganisms and is highly instable. On the first 2 days of life, the GIT is dominated by Firmicutes representing around 60% of the bacterial communities (39). But the low microbial abundance and diversity of the microbiota at that time of life facilitate the potential colonization of external bacteria. For example, a highly conserved phenomenon among animal species is the presence of oxygen within the GIT during the first days of life, promoting the colonization of obligate and facultative anaerobes (72). Most Proteobacteria and Bacteroidetes fall into these categories and were shown to be among the earliest colonizers members in the neonatal gut as it is filled with oxygen. By consuming oxygen, and lowering redox potential (which is positive at birth), it has been speculated they play a key role in preparing the gut for further colonization of strict anaerobes, later required for healthy gut function (31, 62, 73). Rapidly, the proportion of aerotolerant bacteria decreases in the puppy's gut. While Bacteroidetes represents <1% of all sequences analyzed on the first 2 days of life, it represents around 37% of the sequences on the third week, making it the predominant phylum at that period (25, 39).

On the opposite, while it was initially dominating the gut at 2 days of age, Firmicutes shows a high decrease in its relative abundance during the first weeks of life, with the genus *Clostridium* going from 10% of total sequences identified in 2 days old puppies, to 1% at 3 weeks (25, 39). Yet, despite this overall decrease of Firmicutes relative abundance, the abundance of *Lactobacillaceae* in the puppy gut follows a 100-fold increase (25). Combined with the ability of these bacteria to digest milk oligosaccharides and produce lactate, it is suggested that this increase in abundance in the puppy gut is not only linked to oxygen homeostasis, but also to the ingestion of milk by the puppy through the neonatal period. *L. johnsonii* specifically was only found in young puppies (38, 39, 74). Strains of these bacteria are used as probiotic in humans to help promoting antimicrobial properties and reducing proinflammatory activities (28), suggesting a similar role in the gut stability of newborn puppies. In the end, the mean proportion of aerotolerant bacteria keeps decreasing over the neonatal period to represent less than half that of anaerobic bacteria around 2 months (25, 39–41).

It has also been shown that the bacterial richness increased significantly from 2 days to 21 days of age, and the microbial communities clustered separately between those two time points (39). This information showed that important shifts of the bacterial populations of puppies' gastrointestinal tract happen during the first weeks of life, even before the puppy starts eating solid food. Those microbial shifts, and thus the biological properties of the microbiome, are mainly induced by metabolic neonatal events such as the progressive consumption of oxygen in the gut, or the growing ability of the intestine to absorb nutrients, produce bile acids, and develop immune functions (25).

### Changes in the microbiota induced by weaning

Weaning in the dog is described as the progressive transition of the juvenile diet from milk to solid growth diet (like kibbles), usually taking place around 3 weeks old and ending around 8 weeks old, when the puppy is separated from the mother and has no access to milk anymore. Weaning marks an important step in the establishment and development of puppies' gut bacterial population, as the arrival of a new type of food in the GIT promotes the abundance and activities of certain bacterial groups (25, 39, 40). As explained previously, Bacteroidetes went from <1% of abundance at Day 2 to 39% at Day 56 (39) (Table 2), and keep increasing up to adulthood (36, 73, 74). Bacteroidetes, dominated by *Bacteroides* in dogs' gut, are primary degraders of polysaccharides, which is essential for puppies after weaning, as their diet starts to consist mostly of industrial dry petfood, rich in complex carbohydrates. While not as important as Bacteroidetes, Fusobacteria also see its relative abundance growing after weaning, mainly driven by the abundance of *F. perfoentes*, positively correlated with the age of the dogs (36). As mentioned previously, Fusobacteria, can ferment protein and amino-acids to produce SCFAs and branched-chain volatile fatty acids (77). It can be hypothetized that Fusobacteria abundance increased in relation with the consumption of meat products after weaning. Finally, while Firmicutes remained one of the most abundant phyla after weaning, a lot of variation occurred inside the phyla. As seen previously, abundance of some members of *Clostriadaceae* and *Lactobacillus* decreased, like *C. perfingens* and *L. johnsonii*, while the one of others increased, like *C. hiranonis, Faecalibacterium* and *L. animalis* (36, 39). It is likely that bacterial communities keep diversifying after weaning and replace bacteria essential for milk digestion with others having a more essential role in the digestion of complex diets.

**Table 2:** Methodologies and material used in the different studies on growing dog microbiota.

**Study**	**Animals**	**Lifestyle**	**Country**	**Breeds**	**Sampling**	**Isolation method**	**Analysis of results**
Buddington et al. (25)	95 puppies sampled once at either first hour of life, 1, 21, 42, or 63 days old	Born and lived in the same kennel; same diet	USA	Beagle	Stomach, colon and small intestine samples collected after euthanasia; non longitudinal	Microbial culture under anaerobic and aerobic conditions (anaerobic blood + tryptic soy agar)	Statistical Analysis System (SAS) v8.0
Guard et al. (39)	30 same puppies sampled at 2, 21, 42 and 56 days old	Born and lived in the same kennel; same diet	France	Bichon Frise, Maltese, Cocker Spaniel, Jack Russel Terrier, Lhassa Apso, Poodle, Shih Tzu, West Highland White Terrier, Labrador and Golden Retriever	Fecal samples collected by rectal swab; longitudinal	454-pyrosequencing, primers 530F and 1100R, V456 region	QIIME + PICRUSt + LEfSe
Masuoka et al. (40)	10 pre-weaning (mean 13.2 ± 1.8 days) and 10 weaned (mean 6.8 ± 0.4 weeks) sampled once	Born and lived in the same kennel; same diet within the same group of age	Japan	Beagle	Fresh feces samples from defecation; non longitudinal	Microbial culture under anaerobic conditions (tryptic soy + Beerens agar) + FASMAC sequencing	EzTaxon used for bacterial identification + EzR for statistical analysis
Vilson et al. (75)	168 same puppies sampled at 7 weeks, 12–13 months and 15–18 months old	Born and lived in the same kennel then moved to different households at 8 weeks; same diet during all study	Sweden	German Shepherd	Fecal samples collected by rectal swabs; longitudinal	454-pyrosequencing, mix of several bar-code primers (76), V123 and V456 regions	QIIME v1.8.0 + SIM-CA-P+ + LEfSe
Omatsu et al. (74)	20 dogs with only 3 puppies between 0 and 1 year old; sampled once	Different households for each dog and various diet	Japan	Toy Poodle	Fecal samples collected by rectal swabs; non longitudinal	Illumina Mi-Seq, F341 and R805 primers, V3-V4 region	QIIME v1.9.1 + EzR
Pereira et al. (73)	12 puppies sampled at 20, 28, 36, 44 and 52 weeks old	Puppies lived in the same kennel and were fed the same diet within the same group of study	Portugal	Beagle	Fresh feces samples from defecation; longitudinal	Illumina Mi-Seq, F341 and R806b primers, V3-V4 region	QIIME 2 v.2018.6 + SAS
Blake et al. (41)	53 puppies from 1 to 16 weeks + 33 additional puppies older than 8 weeks; all sampled once	The first 53 puppies were born and lived in the same kennel with the same diet; the 33 additional lived in different households with different diets	USA	Golden Retriever, Labrador Retriever and Golden Labrador mixed-breed.	Fresh feces samples from defecation; non longitudinal	Individual qPCR assays for 16S rRNA gene for specific bacteria and F341 and R534 primers, V3 region for universal bacteria sequencing.	Analyses directly made on log DNA + JMP Pro v8
You and Kim (36)	96 dogs of which 16 were between 0.5 and 1 year old; all sampled once	Puppies lived in the same kennel and were fed the same diet	South Korea	Greyhound, Dachshund, Maltese, Bichon, Yorkshire terrier, Chihuahua, Pomeranian, Poodle and Bulldog	Fecal samples collected by rectal swabs; non longitudinal	Illumina Mi-Seq, F341 and R785, V3-V4 region	QIIME 1.9 + LEfSe
Tal et al. (42)	63 puppies (42 healthy and 20 with fading puppy syndrome); sampled once at day 1 or day 8	Puppies were born and lived in 4 different kennels (no information about distribution per kennel and diet)	Unknown	Border Collie, Pembroke Welsh Corgi, Australian Corgi, Australian Shepherd, American Pit bull Terrier, Cane Corso, Cavalier King Charles, German Shepherd, Shih Tzu, Maltese, Caucasian Shepherd, Bernese Mountain Dog, Shetland Sheepdog	Fecal samples collected by rectal swabs; non longitudinal	Illumina Mi-Seq, V4 region, F515 and R806 primers	QIIME 2 v.2019.4 + ALDEx2

### Stabilization of the microbiota after weaning

**Figure 1:**
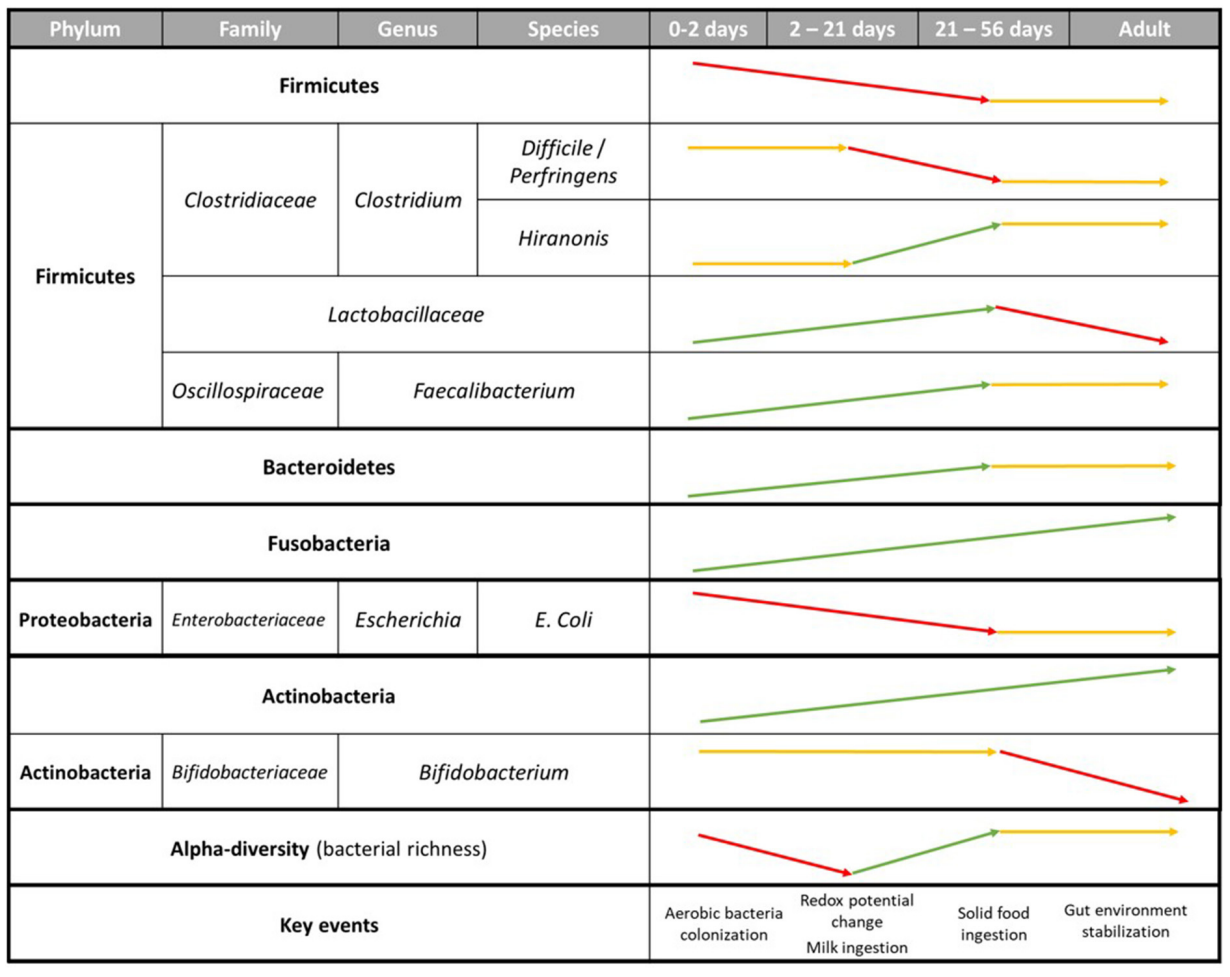
Summary of the evolution of main fecal bacterial groups in puppies from birth to adulthood.

**Figure 2:**
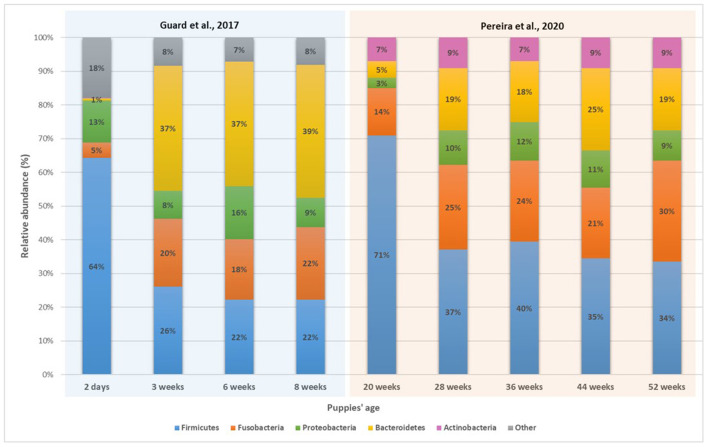
Relative abundance of the main bacteria phyla in puppies' fecal microbiota with age (39, 73).

Once microbial shifts induced by the new diet settle, microbiota composition starts getting more stable following the aging of the puppy, as most of the major factors inducing microbial shifts (oxygen homeostasis, diet transition, environmental changes) already took place. Thus, while microbial species richness of puppies' feces increased significantly from the age of 2 days up to 52 weeks (39, 73, 78), few, or even no changes of microbial diversity have been observed in dogs between 3 months to 12 years old (36, 74, 75, 79). Yet, the composition and diversity of bacterial communities of 7 and 8 weeks old puppies still remain different from dams' one, implying the gut microbiota still had room to develop, along with its host. In humans, it was shown that alpha-diversity increased during youth before stabilizing around the age of 40 years and slowly decreasing in seniors (80–82). Similar to humans, this might suggest the biodiversity of puppies' microbiota increases during the post-natal period, stabilizes a few months after weaning then slowly decreases once senior (Figures 1, 2). This decrease of the gut microbiota richness with aging was mainly witnessed by shifts in the minor taxonomic bacterial ranks (79).

## Other factors shaping the gut microbiome of puppies over time

**Figure 3:**
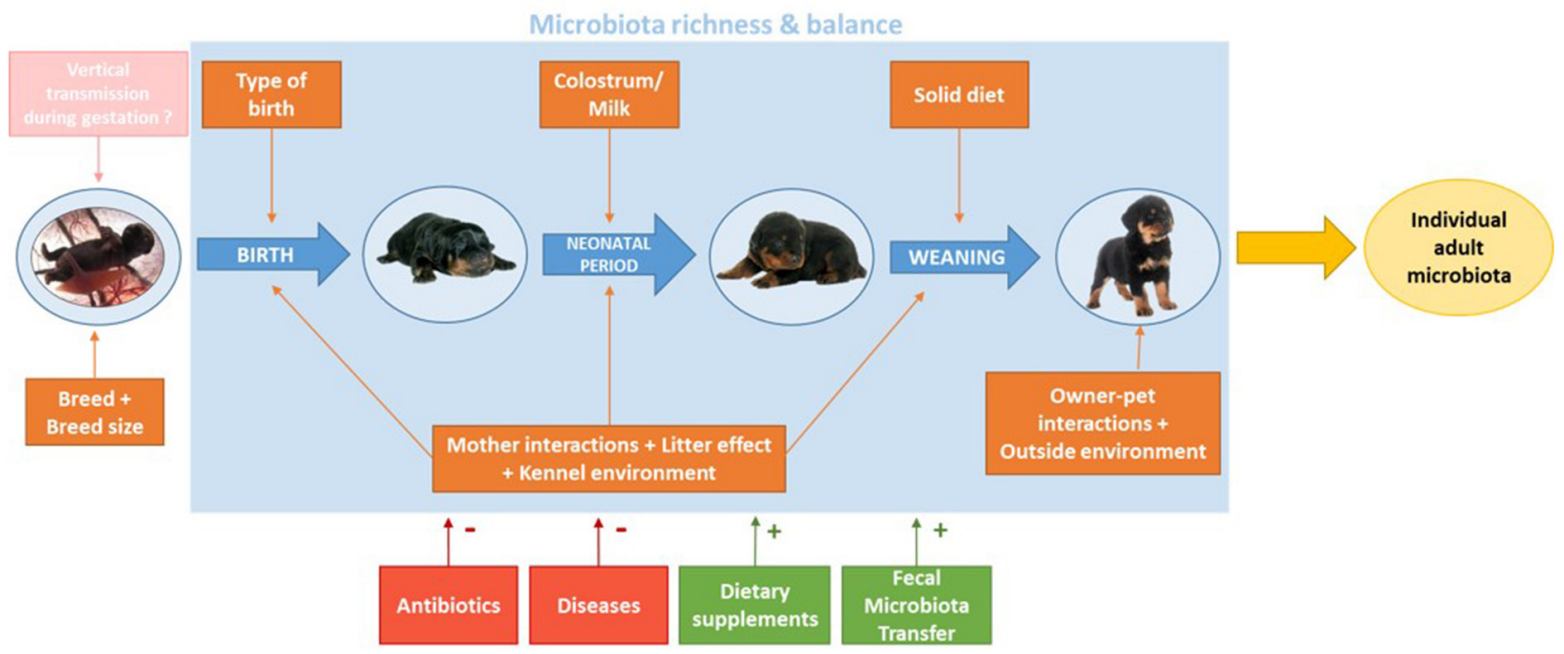
Major factors shaping the development of growing puppies' gut microbiota from birth to adulthood. Orange boxes show obligate factors, while the pink box illustrates a hypothetical impact. Factors in red boxes are facultative factors that can be associated with dysbiosis, while green boxes are facultative factors with beneficial effects on microbiota balance.

Early colonization of the GIT is influenced not only by the physiological status of the animal, but also by a combination of maternal, social, environmental, and dietary factors, each happening at different times during the growth of the puppy, including intra-uterine growth. The role of those factors is to shape the development of the puppy's gut microbiota and create a stable, balanced and unique microbial profile, adapted to the environment the dog grew with (Figure 3) (39).

### Individual characteristics

If some of the factors shaping the gut microbiota can be modulated during one host's life, like diet and environment, some are inherent to dogs even before their birth. This is the case of morphologic and genetic traits, like breed and size. It has been shown that adult dogs microbiota tended to cluster together according to small or large breed size, with *Faecalibacterium* and *Bacteroides* being significantly more abundant in small breeds and *Colinsella* and *Lactobacillus* significantly more abundant in large breeds (83, 84). Such a difference in the microbiota diversity was observed also in small *vs*. large breed puppies from 42 days of age, but not before (39). Since large breed puppies are at higher risk of diarrhea (18), one could hypothetise a relationship between the gut microbiota colonization process and the risk of digestive troubles in large breed puppies. However, this link remains to be investigated.

Studies specifically exploring the difference of fecal microbiota in dogs based on their breed found no difference in alpha or beta diversity between breeds, but differences in the microbial composition. For example, it was observed that Fusobacteria was the dominant phylum in Maltese, while in Poodle, Firmicutes and Actinobacteria were more abundant, even when housed under the same conditions and receiving the same diet (36, 85). Yet, with most of the dogs observed in these studies being older than 1-year-old, the effect of the breed on gut bacterial profile of growing dogs still needs more exploration.

### Before weaning

After its birth, the first step in the modulation of the gut microbiota of the puppy comes from vertical transfer from its mother, occurring as soon as the puppy is born, and possibly even before, during gestation. For example, pregnant bitches share *Bifidobacteria* of their intestinal tract with their offspring (86). In pig, maternal microbes (from milk, skin, vagina, feces) contribute to around 90% of the small intestine bacteria of neonates under 35 days of age (87). These findings support that vertical transmission from mother to their offspring plays a decisive role in shaping the early composition and diversity of the newborn microbiota.

#### Birth mode

Recent studies on infant microbiota suggested that transfer of bacteria from mother to infant is highly dependent on the type of birth, with infants born from cesarean section having an altered microbiota and, as a consequence, a higher risk of health disorders (88, 89). Similar findings were observed in canine studies with lower bacterial diversity in the meconium from cesarean born puppies compared to vaginal born ones, and with higher abundance of potentially pathological bacteria such as *Haemophilus, Streptococcus pluranimalium* or *Rhodotorula mucilaginosa* (38, 90). Furthermore, meconium of puppies after vaginal delivery were shown to be colonized by *Staphylococcus* species almost immediately after parturition (91), with *Staphylococcus* being a common bacteria of the mother vaginal microbiota (38). Alike in human medicine (92, 93), the lower diversity and early colonization of opportunistic bacteria observed in cesarean born puppies might had an impact on their health during the neonatal period. Indeed, it was shown that puppies born by vaginal birth gained weight significantly faster than the ones born *via* cesarean section and puppies presenting a bacteria-colonized meconium gained significantly more weight over the third and fourth days of life than sterile ones (38, 90).

#### Milk ingestion

While the main functions of maternal milk are to bring the energy and immunity required for the survival of the newborn, milk is also a main actor in the colonization of the neonatal intestinal microbiota, as demonstrated in piglets (87). In the dog, 15 different bacterial genera were isolated from colostrum samples, with *Staphylococcus, Kocuria, Enterococcus, Lactobacilli, E. coli* and *Proteus spp*. being the most abundant (90, 94). Among these bacteria, *Lactobacilli* have been demonstrated in human infants to be playing a functional role in the fermentation of specific milk components, like oligosaccharides (95). Indeed, milk oligosaccharides can promote the adhesion of beneficial bacteria to the mucosa, and play a role in modulating inflammation and immunity of neonates (96). Yet, the composition of the milk's microbiota can be altered depending on the type of delivery, as bitches going through a regular birth had greater bacterial richness in their colostrum compared to bitches having cesarean section (90). Bacteria found in dams' milk were also present in newborns' gut, meconium and feces and puppies born vaginally had the same bacterial isolates in their meconium as those identified in their mother's colostrum. Nevertheless, the origin of the gut microbiota in the colostrum is unclear. In humans, similarity of the infant specific oral microbiota to the milk microbiota supports the hypothesis of a retrograde inoculation of the milk, with bacteria (mostly *E. coli*) being transferred from the oral cavity of the newborn to the content of mammary glands (97). But newborn gut and fecal associated bacteria have also been found in human milk (98), which supports the hypothesis of an entero-mammary pathway. This pathway implies that cells of the intestinal lymphoid tissue travel to mammary glands through the lymphatic system and blood, transporting with them maternal microbiota into the milk. Thus, it is highly suggested that part of the initial bacteria colonizing the gut of newborns puppies would be a mix of the gut (*via* milk) and breast skin bacteria from their mother.

#### Litter effect and contact with the dam

In top of milk, dams also impact the microbiota of their progeniture *via* vertical transmission of their own microbiota through physical contact. This direct transmission most probably starts in the dog as soon as the amniotic membrane has been broken. When the dam tears the membrane, cuts the umbilical cord with her teeth and actively licks the newborn, she exposes the newborn to her oral microbiota (91). Being nursed and in contact with the same dam, brethren puppies are exposed to the same maternal bacteria, playing a role in the shape of their initial microbiota composition. In one study, puppies from the same litter had a more similar bifidobacterial population when compared to pups from different litters (26). Vilson et al. (75) also highlighted that puppies at 7 weeks of age, housed together since their birth showed a very close microbial profile, determined by their beta diversity, showing the long term impact of dams on the microbiota composition of their offspring. This litter effect, while still present, was less obvious at 13 and 18 months of age after puppies lived in separate environments, although fed the same diet. The similitudes in the microbial communities of puppies from the same litter may be explained by milk ingestion (identical for all puppies from one given litter), coprophagic behavior and skin contact, among the littermates and their mother before adoption. Before being separated from their mother, maternal factors are the main element shaping the microbiota composition of puppies. Hence, puppies from a same litter tend to have close microbiota composition, and it is when they start having different environments and diets, usually following separation from the mother, that they shape a more specific individual microbiota.

### After weaning

The separation of the puppy from its mother *via* adoption brings a lot of changes in its lifestyle that can induce shifts in the microbial communities.

#### Human contact

After being separated from their mother, most puppies end up living in a close relationship with their new owner. This induces new microbial exchanges between them following their new long-term cohabitation. Human-pet pairs with a close relationship were more likely to share bacteria, mainly *S. intermedius, E. coli, E. faecalis*, and *Acinetobacter lwoffii*, than pairs with a more distant relationship (99). Similarly, it was shown that in households where humans were carrying extended-spectrum cephalosporing-resistant *Enterobacteriaceae*, same strains were also found in dogs, indicating a transfer between humans and dogs (100). While those studies were performed on adult dogs and not puppies, it is highly likely that the same horizontal transfer of bacteria between human and pets can occur independently of the age of the hosts. This suggests that a puppy living alongside a human can see its GIT microbiota shaped by interactions between the two, especially since the gut microbiota of the puppy, as seen previously, is more sensitive to changes during its development.

#### Geographic localization

Vilson et al. showed that the geographic localization matters on the intestinal microbial development of the dog, where it mainly affected the diversity of bacterial populations (75). After leaving the kennel where they lived with their mothers, puppies living in big cities during their first 1.5 year of life had a higher bacterial diversity compared to dogs living in smaller cities or in the countryside. During leash-walks, puppies living in big cities are often exposed to a higher diversity of environments, like parks, streets, buildings, and a wider range of microbes due to more potential exchanges with other leash-walking dogs and people, potentially explaining a higher intestinal bacterial diversity. This impact of the environment on puppies' microbiota composition was also observed after the introduction and intensification of leash-walks outside the facilities. Twenty weeks old puppies (prior to any intense walking) had significantly different alpha and beta diversity than older dogs (from 28 to 56 weeks) (73).

#### Diet

Although a major shift in some gut bacteria families happens at the beginning of weaning, studies evaluating the impact of diet on weaning or growing puppies are still missing. Since the microbiota of puppies is more sensitive to variation than adult ones, it can be assumed that changes of the gut microbiota induced by nutrition on adult dogs also apply to weaned puppies. For example, fecal *Lactobacillus spp., Faecalibacterium* and *Clostridium* abundance was increased in adult dogs fed with higher fiber diet, through beet pulp addition, while that of Fusobacteria, Actinobacteria and Proteobacteria ones decreased (101–103). As for raw or high meat diet, it was observed that dogs had higher abundances of *Lactobacillus, Enterobacteria*, Fusobacteria and *Clostridiaceae* and lower ones of *Prevotella* and *Faecalibacterium* (55, 104–106). In adult dogs, microbial shifts were shown to reverse when dogs are introduced back to their previous diet (105). Yet, since the microbiota of juveniles dogs is more sensible to change and because some shifts in the early stages of life are known to have long term effect on the microbiota composition, further studies would be needed to confirm if puppies possess the same gut microbiota plasticity. In any case, this transition would not be instantaneous, hence the requirement for puppies to have a long diet transition over several days.

## Relationship between puppy's health and gut microbiota

**Figure 4:**
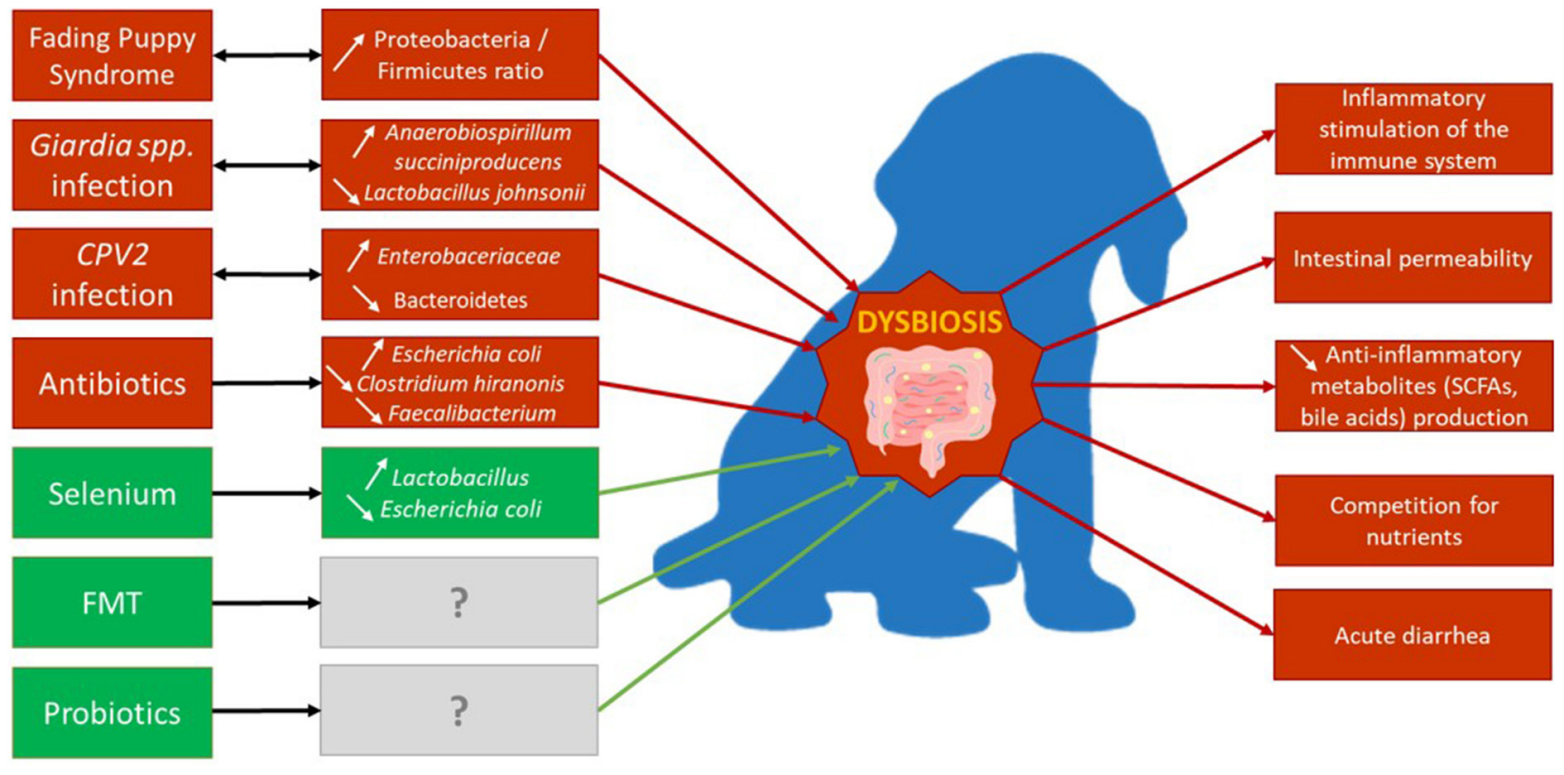
Major factors of dysbiosis in growing dogs' gut and their effects on microbial composition. Red boxes and arrows indicate factors inducing dysbiosis and its consequences, while green ones may participate in decreasing dysbiosis. Gray boxes indicate an unknown effect on microbiota composition.

While it is still unclear if the gut microbiota composition shapes the health status of puppies, and/or the opposite, there is no doubt that a strong correlation exists between the two of them. A summary of the factors related to dysbiosis in puppies and their consequences on the gut microbiota composition are compiled in Figure 4.

### Puppies and dysbiosis

It is actually a complicate task to describe a precise “healthy microbiota.” Indeed, some strains of bacteria are known for their beneficial impact on the infant health, such as *Bifidobacteria*, since low abundances are associated with infant diseases, yet the causal association remains unclear (59). For this reason, a healthy microbiota is usually describe as a balanced homeostatis in the gut of the host (eubiosis), between healthy and potentially pathogenic bacteria. When sudden alterations occur in the composition of the gut microbiota, this balance is broken leading to changes in metabolic activities (i.e., decrease of the production of SCFAs, bile acids and amino acids, oxidative stress, cytokine production…) and making the gut more vulnerable to opportunistic pathogens. This imbalance in the gut microbiota, called “dysbiosis,” is implicated not only in many gastro-intestinal diseases such as inflammatory bowel disease or acute diarrhea (107, 108), but also in systemic disorders, such as diabetes in humans (109). Those disorders are easier to study than eubiosis as they are often linked to diseases with clear phenotypic consequences as opposed to a healthy state. For this reason, literature concerning the impact of microbiota on puppies' health is more focused on negative effect than beneficial ones. Yet, dysbiosis is an evolving concept, and it is still unclear if shifts in the microbiota lead to intestinal diseases or if the diseases themselves are the cause of those microbial changes.

A canine microbiota dysbiosis index (DI) was created to describe, through a unique value, the gut health of an adult dog based on the abundance of some key bacterial families (110). In adult dogs, a value below 0 means the gut microbiota is in a “healthy” state, while a DI of 0 or above indicates gut dysbiosis (53, 56, 107, 111). DI can be calculated in puppies, and healthy puppies from 1 to 6 weeks had a significantly higher DI than adults with an average index of 6 vs. −4 for adults (41). All seven bacteria species involved in the calculation of the score had significantly different abundances in younger dogs compared to adult ones, even though they are healthy, mainly *E. coli, Faecalibacterium*, and *C. hiranonis* (25, 39, 41). Around9weeks old, the abundances of those bacteria started to closely resemble the ones of adults, with an index below zero. This implies the calculation of dysbiosis index, set for adult dogs, might not be suited to assess health disorders in puppies. Due to the dynamic evolution of puppies' microbiota with age, the setting of a puppy's DI would probably require a calculation per week and redefinitions of thresholds.

Several diseases and health conditions were shown to lead to dysbiosis in the gut of puppies such as fading puppy syndrome (FPS) or parasitic and viral infections.

### Fading puppy syndrome

The FPS is a lethal condition describing any puppy born healthy but gradually “fading” and dying within the first 2 weeks of life, with no or very few apparent clinical causes (42, 112, 113). Feces of puppies presenting FPS were shown to have altered beta-diversity compared to healthy puppies, with the Day 1 rectal bacterial beta diversity of puppies being significantly associated with occurrence of FPS later on (42). This difference in fecal microbial community reflected an increase of the Proteobacteria/Firmicutes ratio, with an increased relative abundance of *Pasteurellaceae*, and decreased relative abundance of *Clostridia* and *Enterococcus*, all being positively associated with FPS. Because the exact mechanisms of FPS are unknown, the explanation of how a shift of those bacteria abundances actually impacts the health of puppies, or if FPS is rather a consequence of the shift, is unclear. Those results showed the gut microbial composition as a promising potential biomarker of the newborn dog health to prevent diseases and improve puppies' wellbeing and survival.

### *Giardia* infection

Many enteric parasites were shown to induce significant alterations of the gut microbiota of dogs, with *Giardia*, an ubiquitous intestinal parasite responsible for diarrhea (114), having the most pronounced ones (115). Naturally infected 9-week-old puppies with high fecal load of *Giardia intestinalis* had a higher bacterial richness compared with low cyst load puppies. The opposite was observed in older puppies (about 22 weeks of age), with a reduced fecal bacterial richness of high cyst *vs*. low cyst load puppies (78). Moreover, *G. intestinalis* cyst shedding was positively associated with abundances of many bacterial communities observed in gut diseases in humans, such as *Prevotella* and *Anaerobiospirillum succiniproducens*; these bacteria induce the fragilisation of the mucus of the intestinal barrier. This fragilisation makes it easier for *Giardia intestinalis* to cleave the barrier and allows more enteric pathogens to colonize the gut (78, 116). Finally, in the 22 weeks old puppies, a high cyst load of *Giardia* was also correlated with a decrease in *Lactobacillus johnsonii*. As mentioned previously, this bacterium is specific to young dogs, and probably plays an important role in the early development of puppies gut health thanks to immunomodulation, pathogen inhibition and epithelial cell attachment properties (40, 117, 118).

### Viral infection

Canine parvovirus (CPV2) is one of the most common pathogens affecting dogs, responsible for weaning diarrhea, hemorrhagic enteritis, and death in puppies (119). In one study, four puppies, naturally infected with CPV2 at 6 weeks of age, developed severe gut microbiota alteration with an increase in Proteobacteria abundance, mainly *Enterobacteriaceae*, and a decrease in Bacteroidetes and Fusobacteria abundance (120). Similar shifts were also observed in previous studies with adult dogs presenting inflammatory bowel disease or in puppies infested with *Giardia* (53, 56, 78), suggesting that abundances of these bacterial communities witness a dysbiosis in the dog. Interestingly, those bacterial shifts in CPV2 positive puppies were not permanent. Indeed, 2 weeks following the infection (once recovered from clinical parvovirosis), the microbial composition of the infected group switched back to a composition similar to the non CPV2 infected group (120).

As for many other cases of dysbiosis, it remains unknown to date if the infection with *Giardia* and CPV2 are the cause of those microbial alterations, or the opposite. Indeed, many puppies are positive to both enteropathogens, but not all of them develop clinical signs of infection (diarrhea) (18). One could hypothetise that the intestinal microbiota could be protective in some cases of infections with enteropathogens, either with beneficial bacteria inducing direct competition with pathogens, or by promoting the bacterial production of inhibitory molecules.

### Medical treatments and their impact on puppies' microbiota

#### Antimicrobial and antiparasitic treatments

When facing with diarrhea or chronic gastrointestinal diseases in puppies, antibiotics are the fundamentals of first-intention treatment, and among them, metronidazole is the most prescribed one regarding acute diarrhea in dogs (121). In adult dogs, administration of metronidazole has been shown to disrupt the diversity of the gut microbiota, with an unusual decreased in the abundance of Bacteroidetes, Fusobacteria and Clostridiales, of which important SCFAs producers such as *Faecalibacterium*, and an increase in *E. coli* (111, 122). Yet, most of the abundances of the disturbed bacteria return to baseline levels, after a minimum of 2 weeks following the end of the administration (122). To this date, no similar studies were performed on puppies.

As mentioned earlier, when it comes to parasitic infections, dog breeders usually have to deal with *Giardia* species. In order to slow and eliminate the colonization of this parasite, the most common antiparasitic treatment used is fenbendazole. To date, only one study observed the effect of fenbendazole on the microbiota gut composition of dogs and no alteration of the gut microbiota composition was observed (123). While only a few of the included dogs were puppies (in this case, younger than 10 weeks), this study did not allow to dress a precise impact of antiparasitic treatment on puppies' gut microbiota.

#### Fecal microbial transplantation

Recently, the fecal microbial transplantation (FMT) has been suggested as a therapy in bowel diseases in humans as well as in dogs (111, 124). This therapy consists in transferring the intestinal or fecal content of a healthy donor to a sick individual in order to substitute the dysbiosed microbiota by the healthy one and thus to improve gut health. Dogs treated with FMT recovered faster from acute diarrhea, presented lower abundances of *E. coli* and *Streptococcus spp*., a more diverse gut microbiota and a decreased DI value, than dogs treated with metronidazole (111). Only a few studies addressed usage of the FMT in puppies (125, 126). Pereira et al. did find that treatment with FMT allowed a faster resolution of diarrhea induced by CPV2 compared to puppies treated with antimicrobials (126). Though, the gut microbiota composition was not analyzed in this study. One study evaluated the microbiota profile of puppies (approximate age, 6–8 weeks) transplanted with the fecal microbiota of their mothers (125). It was found the gut microbiota of the puppies did unexpectedly not resemble the maternal one after transfer. It was suggested the microbiota of puppies outcompeted the one of their mothers, but it might also be possible the microbiota needed more time to mature depending on the window of time of the study. Indeed, one species, *P. copri*, was still found in both mother and FMT puppies, and not in non FMT puppies. Yet it is important to keep in mind FMT's expected effects are dependent of many factors, like the age of the donors and recipients, their physiological state or the desired effect, and more studies are needed to fully understand its mechanisms and the impact on microbiota profiles.

#### Dietary supplementation and probiotics

Lastly, other than medication, gut microbiota can also be modulated with dietary supplements, such as minerals or probiotics. The effect of a selenium supplementation on the gut microbiota and health of puppies was assessed in a recent study (73). Selenium is a trace element with antioxidant properties which is known to reduce intestinal inflammation, allowing to create an adequate environment for the gut microbiota development. This modulation of the gut microbiota increases the efficacy of the intestinal barrier and immune responses (127). When administered to puppies (from 20 to 52 weeks of age), organic selenium led to a lower abundance of *E. coli* and a higher abundance of *Lactobacillus*, which itself provoked a higher concentration of lactate in feces. This supplementation also increased the production of volatile fatty acids, mainly butyrate and propionate, being used as energy source or taking part in immunomodulatory properties. Yet, selenium only represents one type of dietary supplement among the many other existing. Thus, the exact impact of such microbial shifts on health of the growing dog fed with dietary supplementations requires further investigations.

Probiotics are defined as “live microorganisms which when administered in adequate amounts confer a health benefit on the host” (128). Most of the probiotics used in humans and animal research are lactic acid bacteria and *Bifidobacteria* strains (129), with effects ranging from modulation of the immune system, protection from enteropathogens, stimulation of growth and regulation of obesity (130). Although some of these studies were conducted in adult dogs (131, 132), very few studied the impact of probiotics on the gut microbial composition, especially in puppies (133, 134). In one study, administration of *Lactobacillus johnsonii NCC533* strains to dams, from the end of gestation (3 weeks prior to parturition) to the end of lactation, and their puppies, from 3 to 8 weeks of age, did not influence the fecal microbiota composition of the puppies when compared to non-supplemented ones (75). Yet, another study showed that dogs (*n* = 5) supplemented with lactic acid bacteria strains induced alteration of indigenous *Lactobacillus* and their dominance in the jejunal chime (131). These promising results need to be confirmed through additional studies to dress a more precise overview of the impact of probiotics on the microbiota of puppies and the benefits for their health.

## Discussion

Many progresses have been made in the field of puppies' microbiota, with 18 articles published between 2019 and 2021 against only 8 between 2010 and 2019 (135). This study aimed to review those recent advances to identify which factors influence microbiota colonization in the growing dog. Evidence based information are necessary to advise dog breeders and owners in order to promote healthy microbiome, and thus healthy lifestyle, in puppies. Yet, knowledge remains limited, as most studies involving dog microbiota focus on adult individuals rather than puppies, with very few data available on dogs younger than 4 weeks old.

### Recommendations to promote a healthy and balanced microbiota for puppies

Based on the reviewed information, aging, weaning, type of birth, maternal factors, environment and overall health are the main factors to shape the microbiota during puppies' growth. More precisely, natural birth and maternal colostrum intake seem to be crucial parameters in the initial development of the puppy's microbiota, as they are the first factors to shape the newborn microbiota. Vaccination (particularly against CPV2) and antiparasitic drugs administration (particularly against *Giardia* spp.) are to be advised to dog breeders in order to decrease the risk of dysbiosis, potentially leading to morbidity in young dogs. Later on, when puppies get older and start eating solid food, their microbiota shows one of the biggest shift, due to the new nutrients ingested and the metabolic properties they lead to (fiber, starch and meat digestion), yet those changes follow an adaption of the microbiota to the new diet and are not linked to health or disease conditions. Leash-walks are then recommended to favorise bacterial exchanges with the environment and other individuals, increasing the diversity of the gut microbial population of the puppy. Despite no studies have been performed on puppies, antibiotic administration may have, as demonstrated in adult dogs, a strong impact on the gut microbiota, with rapid and significant drops in taxonomic richness, diversity, and evenness (122, 136). For this reason, their administration should be avoided as much as possible to preserve healthy gut microbial communities. All those recommendations would allow puppies to build up a balanced and rich microbiota so that they reach a more stable state once adult. This healthy and diversified microbiota is crucial to balance pro- and anti-inflammatory activities of the gut, preventing excessive inflammation while still being able to promptly respond to infections and pathogens. Key points from this review are summarized in Figure 3.

### The puppy core gut microbiome and interindividual variability

In humans, the gut microbiome reaches a stable state around the age of 3 years (82, 137), while it usually happens in less than a year for dogs (41). The reason might come from differences in metabolism between the two species, and also because human children start eating solid and adult-like food later than puppies do. Before that period, and as shown previously, numerous shifts of the microbiota composition occur. This makes it difficult to describe a “puppy core gut microbiome,” as it might be heavily different depending on the age of the animal. Yet, it is possible to highlight specific taxa and species of bacteria which are abundant in puppies but almost absent in adult dogs and vice versa (Figures 1, 2). While the five main phyla are the same between adult and puppies, their repartition are different. Proteobacteria are more abundant in puppies fecal microbiota while Bacteroidetes and Fusobacteria are more abundant in adults. As for specific taxa, puppies have higher abundances of *Bifidobacterium, Enterobacteriaceae* (mainly *E. coli*), *Lactobacillaceae* (mainly *L. johnsonii*) and some *Clostridiaceae* (*C. perfringens* and *C. difficile*). *Faecalibacterium, L. animalis* and *Turicibacter* on the other hand, are more present in adult dogs.

Another difficulty in defining a puppy core gut microbiome is the fact a great variability of results exists among studies characterizing the dog gut microbiota. For example, two studies observing puppies around the age of 7 weeks, reported a relative abundance of Firmicutes of respectively 22% (39) and 78% (75) of the sequences read. Such differences of composition and abundance among studies tend to indicate that interindividual variability also plays an important role in the intestinal microbiome development during the first months following weaning (125). This individual variability can be an expression of many factors, such as genetic, breed, diet or type of birth, which mixed together, form each puppy own specific gut microbiome. Yet, it can also come from limit in methodologies and studies.

### Limits of studying puppies

Literature analyzed in this review showed some limitations. First, most studies used a relatively small number of animals, with study populations of puppies ranging from 8 to 168 puppies (mean of 46 puppies per study, out of 16 studies involving puppies' microbiota), and several discussing themselves the small size of the included population. Most human studies aiming to describe or characterize the microbiota composition include hundreds of participants, with even more samples collected. This is actually an important point for studies dealing with microbiota as microbial profiles are quite variable, even between individuals living in the same environment, with similar lifestyle, age and diet (138, 139). But unlike humans, large populations of juveniles are hard to obtain with dogs. In order to avoid variability associated with breeds and environment, it is usually preferred to work in a single kennel, at the cost of the number of individuals. To enlarge their populations, some studies used dogs living in households (40, 74), but this implies to select sampling methodologies so that owners can perform the required manipulations without the need of a specialist, and it also means environmental parameters cannot be standardized. Studying newborn puppies is even harder to organize than adult dogs. The exact number of newborns cannot be known until parturition, meaning the number of dams recruited and hence, the number of puppies per litter, must be hypothesized beforehand to have enough statistical power during the study. Furthermore, pregnant dams need to be recruited in a way so that they can all give birth in the same environment and over a limited period of time, to allow standardization. Working with puppies also involves ethical issues when it comes to sampling, due to the close proximity between humans and dogs and the raise of anthropomorphism in science (140).

Another point making the extrapolation among studies limited is the dissimilarity of methodologies from logistic and collection to analysis (see Table 2). Variations concern the site of collection analyzed (rectal vs. feces), the tool used for collection (samples picked from ground or collected before expulsion) and the storage conditions (temperature, buffer, humidity). Sequencing techniques are even more diverse: classical bacterial cultivation (25, 40) vs. next-generation technologies such as 16sRNA amplicon sequencing, with 16sRNA itself declining in different techniques, such as 454-pyrosequencing (39, 75) and Illumina (141). These differences in protocols and techniques may contribute to the high variations of microbiota observed in puppies of the same age (Table 2). On top of methodologies, diet, environment and dog breeds can also impact microbiota results in many ways, as shown in this review. Some studies only observed one specific breed (40, 74, 75), while other studied several breeds at once (36, 39, 41). Also, some dogs were raised in a kennel (36, 39, 73), with limited access to the outside, while some others were living in host families and had access to more various locations through leash-walks, allowing more microbial exchanges with the environment (41, 74, 75).

All those elements need to be taken into account when comparing studies characterizing puppies' microbiota.

### Future research needs

Even though some recommendations could be drawn from this review, studies on gut microbiome and long term health are still vary scarce. Advances in human studies have allowed to link gut microbiota to type 1 and 2 diabetes (14), autoimmune diseases (142), and even mental health (143), but most of those topics remain to be studied in dogs. An interesting topic would be the link between the host cellular metabolism and its gut microbiota. For example, in humans, colonocytes have been shown, in case of oxydative stress, to rise the redox potential of the gut lumen, favorising the colonization of facultative anaerobic bacteria, which include a large spectrum of potentially pathogenic bacteria (28). In the opposite, “healthy” coloncytes would induce an anaerobiose state in the gut by rapidly consuming oxygen through fatty acid oxydation. Such mechanisms can have huge impact on the neonatal and weaning period of the puppies which are more prone to dysbiosis. Some other factors, studied in adult dogs, remain to be explored on puppies, like the impact of antibiotics and anti-inflammatory medications or bodyweight condition of the puppies in the gut microbiota composition. Another complex subject happened to be the relationship between puppys' health condition and microbiota composition. While studies showed disruption in the microbiota composition during health disorders, there is actually no evidence allowing to determine whether the microbiota or the health condition happen to be the cause or consequence of the other. Advances in this field would allow to potentially use the microbiota composition as a tool to prevent some diseases. Since a healthy early colonization of the infant gut microbiota has been shown to have beneficial effect on the later health in humans (144), exploration of intestinal colonization since birth, or even during fetal life, could prove promising to reduce and prevent neonatal and pediatric mortality. The bacterial imprinting from a mother to its puppies hasn't been properly studied yet, while it could prove to be a promising approach to modulate the microbiota of the puppies before their birth, by modulating the one of the pregnant dams. This approach appears to be especially appealing in the case of puppies since around 10% of puppies born alive die over the first 2 months of life (17), with a potential to use microbiota composition as a marker of health to reduce neonatal morbidity.

## Conclusion

The early neonatal period is a critical phase for puppies, during which the gut microbiota develops and modulates a healthy environment in the gastro-intestinal tract with long-term effect on the health of the puppy. This impact of the microbiota on health is dependent of its composition and how it evolves during the early growth. We reviewed many factors which can modify the microbial communities of puppies' gut, of which aging, environment, type of parturition and social interaction are the most important ones. Some factors, like vaginal birth or milk ingestion, are more studied and showed they can promote a healthy microbiota. But other factors, such as antibiotic treatments or overweight, have not yet been studied in a population of puppies, and it remains to be confirmed if the impact on the microbiota composition is the same as in adult ones. In any case, the combination of all those factors shapes the definitive microbiota of the puppy once it becomes adult. The highlighted relationship between the overall health of puppies and their gut microbiota composition could be used as a critical tool to predict the development of diseases. Bacteria with key roles in gut homeostasis could be monitored and used as biomarkers to prevent health disorders and treat them accordingly, with, for example, the use of probiotics. This prevention could even start during gestation by linking the gut microbiota of the dam to the health of the offspring, with possibilities to modulate the initial gut microbiota of the puppy before its birth and reduce neonatal mortality or morbidity risks. In that perspective, the development of new technologies is promising to explore deeper the microbiome of puppies. Indeed, omics techniques have improved mechanistic research and clinical trials in adult dogs to determine the impacts of different factors on the microbiome. Applying multi-omics approach and integrating datasets may assist to identify loss of microbiome functions, vacant functional niches important for puppies' disease prevention.

## Author contributions

QG drafted the review, designed figures, and wrote the final manuscript after reviewing. HM, SC, and EA reviewed and contributed to the writing of the final manuscript. All authors contributed to the article and approved the submitted version.

## Funding

QG was the fellowship of a doctoral grant financed by Région Occitanie Pyrénées-Méditerranée.

## Conflict of interest

Author EA is employed by the company Lallemand SAS. The remaining authors declare that the research was conducted in the absence of any commercial or financial relationships that could be construed as a potential conflict of interest.

## Publisher's note

All claims expressed in this article are solely those of the authors and do not necessarily represent those of their affiliated organizations, or those of the publisher, the editors and the reviewers. Any product that may be evaluated in this article, or claim that may be made by its manufacturer, is not guaranteed or endorsed by the publisher.

## Abbreviations

CPV2: Canine parvovirus type 2
DI: Dysbiosis index
GIT: Gastrointestinal tract
FMT: Fecal microbial transplantation
FPS: Fading puppy syndrome
SCFAs: Short-chain fatty acids.
